# Chrom-Sig: de-noising 1D genomic profiles by signal processing methods

**DOI:** 10.1093/bioinformatics/btaf645

**Published:** 2025-12-01

**Authors:** Nandita J Gupta, Zachary Apell, Minji Kim

**Affiliations:** Department of Electrical and Computer Engineering, University of Michigan, Ann Arbor, MI 48109, United States; Gilbert S. Omenn Department of Computational Medicine and Bioinformatics, University of Michigan, Ann Arbor, MI 48109, United States; Gilbert S. Omenn Department of Computational Medicine and Bioinformatics, University of Michigan, Ann Arbor, MI 48109, United States; Department of Biostatistics, University of Michigan, Ann Arbor, MI 48109, United States; Department of Electrical and Computer Engineering, University of Michigan, Ann Arbor, MI 48109, United States; Gilbert S. Omenn Department of Computational Medicine and Bioinformatics, University of Michigan, Ann Arbor, MI 48109, United States

## Abstract

**Motivation:**

Modern genomic research is driven by next-generation sequencing experiments such as ChIP-seq, CUT&Tag, and CUT&RUN that generate coverage files for transcription factor binding, as well as ATAC-seq that yield coverage files for chromatin accessibility. Due to the inherent technical noise present in the experimental protocols, researchers need statistically rigorous and computationally efficient methods to extract true biological signal from a mixture of signal and noise. However, existing approaches are often computationally demanding or require input or spike-in controls.

**Results:**

We developed Chrom-Sig, a Python package to quickly de-noise 1D genomic coverage tracks by computing the empirical null distribution without prior assumptions or experimental controls. When tested on 19 ChIP-seq, CUT&RUN, ATAC-seq, and snATAC-seq datasets, Chrom-Sig can effectively decompose the data into signal and noise components. Notably, Chrom-Sig performs de-noising and peak calling in 1–2 h using around 20 GB of memory. The de-noised signal corroborates with biologically meaningful results: CTCF CUT&RUN data retained a high percentage of peaks overlapping CTCF binding motifs, while ATAC-seq and RNA Polymerase II data were enriched in enhancers and promoters. We envision Chrom-Sig to be a versatile and general tool for current and future genomic technologies.

**Availability and implementation:**

Chrom-Sig is publicly available on GitHub (https://github.com/minjikimlab/chromsig) and Zenodo (doi: 10.5281/zenodo.17488772) under the MIT licence.

## 1 Introduction

The advances in high-throughput sequencing technologies have enabled researchers to probe various activities and structures in the genome. For example, one can probe gene expression (RNA-seq) (Mortazavi *et al.* 2008), protein binding intensity or histone modification (ChIP-seq, CUT&RUN, CUT&Tag) (Robertson *et al.* 2007, Skene and Henikoff 2017, Kaya-Okur *et al.* 2019), chromatin accessibility (ATAC-seq) (Buenrostro *et al.* 2015), and chromosome conformation (Hi-C, ChIA-PET) (Fullwood *et al.* 2009, Lieberman-Aiden *et al.* 2009). More recently, single-cell and multi-ome methods have provided the unprecedented resolution and information to simultaneously probe multiple modalities in a single cell (snATAC-seq, HiRES) (Preissl *et al.* 2018, Liu *et al.* 2023). Due to the significance of genome function and structures on human health, the National Institutes of Health ENCODE (ENCODE Project Consortium 2012) and 4D Nucleome (Dekker *et al.* 2017, Reiff *et al.* 2022) consortia have led concerted efforts to generate the genomic data in diverse mammalian tissues and cell types.

While these methods have the potential to uncover meaningful biological phenomena, the progress is often impeded by the inherent technical bias and noise present in the experimental protocols. To avoid misinterpreting the data, one needs to apply a rigorous statistical method to assess the significance of observed data compared to the background null model. However, new assays often lack a known theoretical background distribution. One approach to mitigate the problem is to perform input control or spike-in experiments to obtain the experimental background, but doing so can be laborious and costly or infeasible for rare samples.

As an alternative solution, researchers have developed computational algorithms Coda (Koh *et al.* 2017) and AtacWorks (Lal *et al.* 2021) to de-noise 1D ChIP-seq or ATAC-seq data, respectively. However, these and other machine learning methods can be computationally intensive, require a large training set that may be lacking for new assays, or overfit the data. An ideal method should be computationally efficient, statistically rigorous, and biologically interpretable—all without requiring additional wet-lab experiments. Towards this goal, we developed a Python package Chrom-Sig, which quickly obtains empirical background distribution to assign a statistical significance to each observed read, thereby retaining reads with high significance as means to extract true biological signal in any 1D genomic assays.

## 2 Materials and methods

A simple, yet powerful way to probe the empirical null distribution is to place each observed read in a random location within the same chromosome many times and record the signal intensity therein. However, the key bottleneck of this approach is the efficiency: searching for a given interval in a large bedGraph file is a computationally expensive job. This problem is exacerbated by the fact that robust statistics requires a large number of samples. To overcome this problem, we have previously developed pyBedGraph (Zhang *et al.* 2020), which can quickly obtain summary statistics from a 1D genomic signal. Specifically, obtaining the exact mean for 10 billion intervals is estimated to take 43 min with pyBedGraph and 7.4 days with a similar software pyBigWig (Ramírez *et al.* 2016).

**Figure 1.**
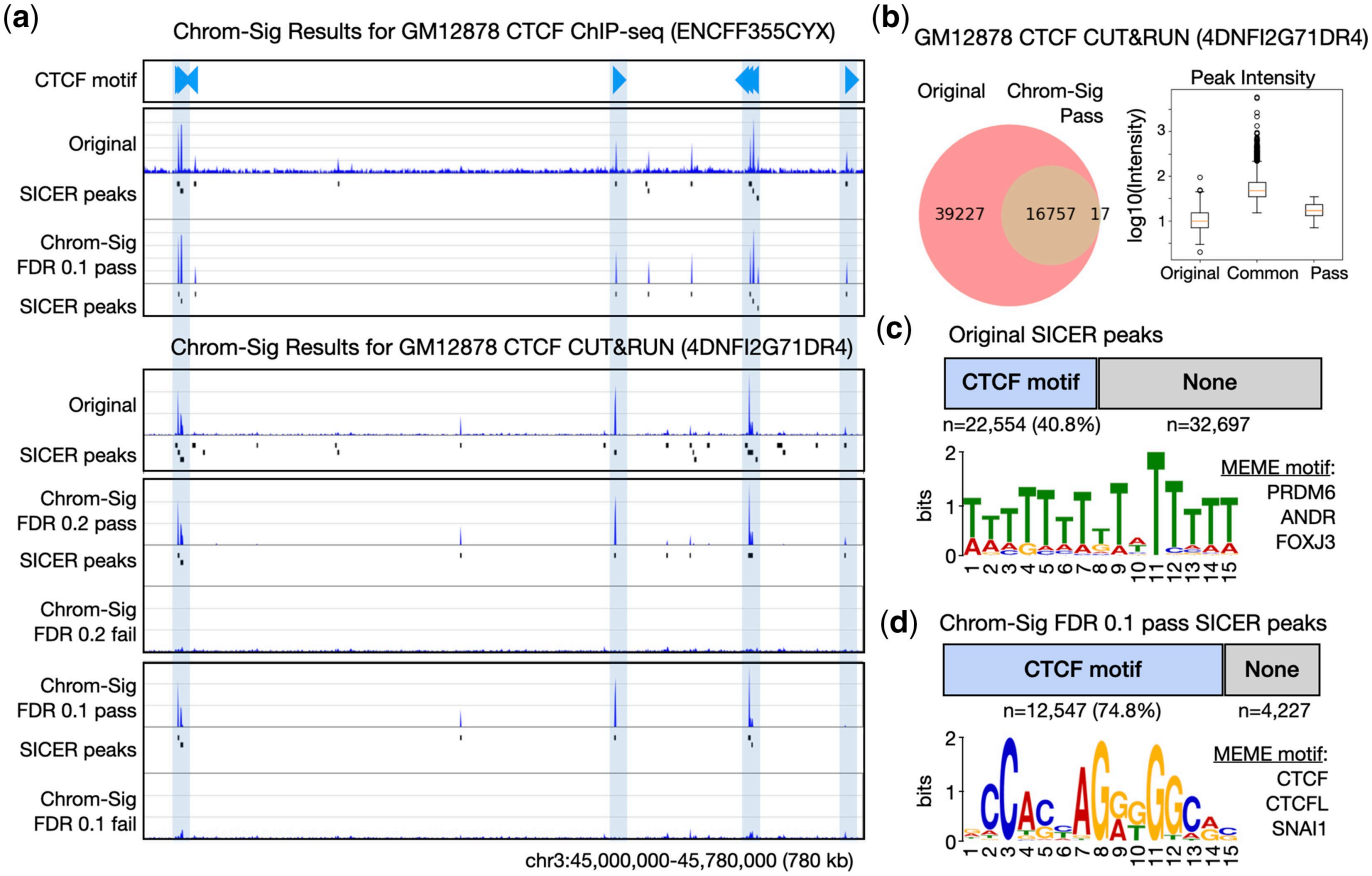
Chrom-Sig results on GM12878 CTCF ChIP-seq and CUT&RUN datasets. (a) Browser views of CTCF binding motifs with orientation (blue triangles) and coverage tracks generated by piling up the original (before Chrom-Sig) and Chrom-Sig ‘pass’ or ‘fail’ reads, accompanied by the peaks called by SICER. (b) Venn diagram of the peaks called on the original and Chrom-Sig ‘pass’ reads pile-up reads using false discovery rate (FDR) of 0.1 and 5000 pseudo-reads, with boxplots of maximum peak intensity for each peak. (c) Number of peaks overlapping CTCF motifs, and top MEME result on the original GM12878 CTCF CUT&RUN data before Chrom-Sig. (d) Similar to panel c for Chrom-Sig ‘pass’ results.

By leveraging pyBedGraph, we implemented the enrichment test—assessing the statistical significance of each observed read by comparing the observed signal to the empirical null distribution in a random location—in a Python package Chrom-Sig (Fig. 1, available as supplementary data at *Bioinformatics* online). As an input, Chrom-Sig accepts BAM or bed files of aligned paired-end or single-end reads generated by any 1D genomic assays including ChIP-seq, CUT&RUN, ATAC-seq, and snATAC-seq. The enrichment test is performed on the coverage bedGraph file generated by piling up reads, resulting in the ‘pass’ or ‘fail’ reads based on the false discovery rate (FDR) given the number of pseudo-reads for the empirical null. The pile-up of ‘pass’ reads further undergo a peak calling algorithm SICER (Xu *et al.* 2014), providing an accurate set of peaks for users. Chrom-Sig is implemented in Python3 using a few strategies to further optimize speed and memory usage. Detailed methods are provided in Methods 1, 2, and 3, available as supplementary data at *Bioinformatics* online.

## 3 Results

We applied Chrom-Sig on 11 paired-end reads and 8 single-end reads datasets (Table 1, available as supplementary data at *Bioinformatics* online) from ChIP-seq, CUT&RUN, ATAC-seq, and snATAC-seq experiments in GM12878 and K562 cell line downloaded from the ENCODE (Sloan *et al.* 2016) and 4DN (Dekker *et al.* 2017, Reiff *et al.* 2022) data portals. All runs were on an Intel^®^ Xeon Gold 6154 CPU @ 3.0 GHz.

We first ran Chrom-Sig with 5000 pseudo-reads (Table 2, available as supplementary data at *Bioinformatics* online). The paired-end data consisted of 7–174 million uniquely mapped reads and FDR of 0.2 retained 11%–76% of them, while FDR of 0.1 kept 5%–67% (Fig. 2a, available as supplementary data at *Bioinformatics* online). Single-end data had between 4 and 15 million uniquely mapped reads, of which 1.5%–35% and 0.5%–30% were kept for FDR of 0.2 and 0.1, respectively (Fig. 2b, available as supplementary data at *Bioinformatics* online). Doing so took between 0.06 and 2.9 h and 2–85 GB of memory (Fig. 3, available as supplementary data at *Bioinformatics* online). To evaluate the effect of the number of pseudo-reads on the runtime and percentage of pass, we varied the pseudo-reads from 50 to 5000 on GM12878 CTCF ChIP-seq and RAD51 ChIP-seq data. As expected, the runtime had a linear trend with ∼100 s on 1000 pseudo-reads, and the percentage of pass reads converged to 14% and 6.8% for CTCF and RAD51 data, respectively, after 2500 pseudo-reads (Fig. 4, available as supplementary data at *Bioinformatics* online).

Chrom-Sig effectively de-noised the data. For example, the GM12878 CTCF ChIP-seq data were originally noisy with a large portion of reads in non-binding sites, but Chrom-Sig with FDR of 0.1 retained only the reads with strong binding (Fig. 1a). Similarly, CTCF CUT&RUN data originally had a high number of false positive peaks due to noise. After running Chrom-Sig with FDR of 0.2 or a more stringent 0.1, the peaks identified by SICER on Chrom-Sig ‘pass’ (signal) were highly specific; the ‘fail’ (noise) pile-up is non-specific and resembles the data from input control experiments (Fig. 1a; Fig. 5, available as supplementary data at *Bioinformatics* online). This general trend is observed for all 19 datasets, where the de-noised data are visually cleaner and the peaks contain fewer false positives than the original data (Figs 6–8, available as supplementary data at *Bioinformatics* online). In particular, the two YY1 ChIP-seq data were highly noisy and of low depth (∼5 million reads), yet Chrom-Sig was able to recover a small number of peaks that coincide with those of two other replicates with high depth (∼15 million reads) (Fig. 8, available as supplementary data at *Bioinformatics* online).

To compare the peaks before and after Chrom-Sig, we computed the proportion of overlap. As visually speculated for GM12878 CTCF CUT&RUN data (4DNFI2G71DR4), there were originally 55 251 peaks, of which 16 757 (42.7%) were recovered by Chrom-Sig; it also identified a small number (17) of new peaks (Fig. 1b). The binding intensity for each group of peaks verified that the common peaks (‘Common’) were the strongest—likely representing true CTCF binding sites—followed by Chrom-Sig-specific (‘Pass’) and Original-specific (‘Original’) groups (Fig. 1b). Similar results are shown for all 19 datasets (Figs 9 and 10, available as supplementary data at *Bioinformatics* online).

We sought to verify that Chrom-Sig retains biologically meaningful signal while removing false positive peaks. Using the known CTCF motif binding sites as a proxy of true signal, we verified that the original SICER peaks had only a small proportion (40.8%) overlapping with CTCF motifs and the top MEME (Machanick and Bailey 2011) motif result showing the enrichment of PRDM6, ANDR, FOXJ3 instead of CTCF (ranked 18) (Fig. 1c). By contrast, Chrom-Sig peaks mainly overlapped CTCF motifs (74.8%) and were strongly enriched in CTCF motifs via MEME (Fig. 1d). Top 3 MEME motifs are also presented (Fig. 11a, available as supplementary data at *Bioinformatics* online). We verified with other GM12878 CTCF ChIP-seq and CUT&RUN data that Chrom-Sig was specific in identifying peaks overlapping known CTCF motifs (Fig. 11b, available as supplementary data at *Bioinformatics* online). Similarly, Chrom-Sig particularly enriched for active promoters and enhancers in ATAC-seq and RNA Polymerase II ChIP-seq data—as expected and desired (Fig. 12, available as supplementary data at *Bioinformatics* online).

Finally, we compared Chrom-Sig to AtacWorks. While AtacWorks de-noises ATAC-seq data at a comparable level as Chrom-Sig (Fig. 13, available as supplementary data at *Bioinformatics* online), it fails to recover meaningful signal for protein binding sites. For example, AtacWorks increased the background noise of CTCF CUT&RUN data resulting in a high number of peaks (*n* = 1 77 485), many of which contain false positives as evidenced by low precision (0.14) and inability to capture CTCF binding motifs (Fig. 14, available as supplementary data at *Bioinformatics* online). Similarly, on CTCF ChIP-seq data, AtacWorks struggled to recover CTCF binding sites by calling too few peaks (*n* = 9350) with low recall (0.3), thereby lacking statistical power to identify known motifs (Fig. 15, available as supplementary data at *Bioinformatics* online). Thus, Chrom-Sig is appropriate for de-noising a wide range of experimental data including those probing open chromatin regions and protein binding sites.

## 4 Discussion

We developed Chrom-Sig and demonstrated its ability to de-compose 1D genomic data into signal (‘pass’) and noise (‘fail’). Specifically, de-noising the data with Chrom-Sig resulted in highly specific peaks that correlated with biological evidence such as CTCF binding motifs and active chromatin states. However, one drawback of the current version is the lack of guidance on FDR threshold and the number of pseudo-reads. We generally recommend FDR of 0.2 and 5000 pseudo-reads, as a stringent FDR may unintentionally remove signal and low pseudo-reads may not provide enough statistical power. As the field of genomics continue to develop novel experimental technologies to measure multiple types of biological signal at single-cell level, it will be imperative to distinguish true signal from technical noise. We envision Chrom-Sig to be instrumental in filtering out inherent technical noise without requiring input or spike-in control experiments, thereby providing a versatile and widely applicable tool for researchers.

## Supplementary Material

btaf645_Supplementary_Data

## Data Availability

All data and implementation details of the code can be obtained from GitHub (https://github.com/minjikimlab/chromsig).
