# Supplementary material for: Chrom-Sig: de-noising 1D genomic profiles by signal processing methods
Source: Bioinformatics. 2025 Dec 1;41(12):btaf645. doi: 10.1093/bioinformatics/btaf645 (PMC12715305; doi:10.1093/bioinformatics/btaf645)
Supplement: btaf645_Supplementary_Data [file btaf645_supplementary_data.pdf]

Chrom-Sig: de-noising 1-dimensional genomic profiles by signal processing methods

Nandita J. Gupta[1,2], Zachary Apell[3,2], Minji Kim[2,1*]

[1]Department of Electrical and Computer Engineering, University of Michigan, Ann Arbor, MI, USA.

[2]Gilbert S. Omenn Department of Computational Medicine and Bioinformatics, University of Michigan, Ann Arbor, MI, USA.

[3]Department of Biostatistics, University of Michigan, Ann Arbor, MI, USA.

*Corresponding author. E-mail address: minjilab@umich.edu

Supplementary Method, Tables and Figures

Supplementary Method S1. `Chrom-Sig` algorithm
Supplementary Method S2. `Chrom-Sig` usage and commands
Supplementary Method S3. Analysis of `Chrom-Sig` results
Supplementary Table S1. Details of 19 datasets used in the benchmark
Supplementary Table S2. Runtime, memory, statistics for 5000 pseudo-reads
Supplementary Figure S1. Overview of `Chrom-Sig`
Supplementary Figure S2. Overview of Datasets
Supplementary Figure S3. Runtime and Memory
Supplementary Figure S4. Runtime and Pass % vs. Number of Pseudo-Reads
Supplementary Figure S5. `Chrom-Sig` fail output vs. IgG control
Supplementary Figure S6. Paired-end Reads Example for GM12878
Supplementary Figure S7. Paired-end Reads Example for K562
Supplementary Figure S8. Single-end Reads Example
Supplementary Figure S9. SICER Peaks in Paired-end Reads Data Before and After `Chrom-Sig`
Supplementary Figure S10. SICER Peaks in Single-end Reads Data Before and After `Chrom-Sig`
Supplementary Figure S11. CTCF Motif Analyses
Supplementary Figure S12. ChromHMM State Annotation Distribution
Supplementary Figure S13. `Chrom-Sig` vs. AtacWorks
Supplementary Figure S14. `Chrom-Sig` vs. AtacWorks CUT&RUN
Supplementary Figure S15. `Chrom-Sig` vs. AtacWorks ChIP-seq

Supplementary Method S1. `Chrom-Sig` algorithm

The key idea of Chrom-Sig is to take the observed read and place it on a random location of the same chromosome and compare the coverage between the observed and the expected. Through many rounds of re-sampling, we obtain the p-value by counting the number of occurrences in which the expected coverage exceeds the observed coverage. Input datasets are in bam or bed file format. Chrom-Sig uses a reference genome sizes file as indication for which chromosomes to keep, and generates a new standard bed file, containing only standard chromosomes.

Since each chromosome must be processed separately when de-noising, Chrom-Sig generates a separate node for each chromosome in order to run all chromosomes in parallel. Within each node, the de-noising process generates a list for which chromosomes are in the bed file, and only runs the Python program for de-noising on those specified chromosomes. During this process, it also checks for the coverage of each chromosome. Using bedtools (2.31.1), bedtools summary generates a coverage report on each chromosome in a bed file. By dividing the total interval basepairs (column 4) by the chromosome length (column 2) in the report, we obtain a fraction for the coverage of each chromosome. Only the data in the bed file with more than 20% coverage for a given chromosome is processed. Chrom-Sig de-noising module is written in Python 3.9.

The first step in the de-noising process is to read in the bed file. Instead of reading in all the data, Chrom-Sig only reads in the data for the current chromosome, ignoring the remainder of the file.

The most computationally expensive part of Chrom-Sig's denoising algorithm is generating and processing pseudo-reads, which are random placement of observed reads in a given chromosome. Note that pseudo-reads are not simulated sequencing reads. For every observed paired-end read $[[c_1,\ s_1,\ e_1],[c_2,\ s_2,\ e_2]]$ corresponding to the chromosome, start, and end positions of the read (note that $c_1 = c_2$), we create the following distance tuple:

$$((s_1 - s_1),(e_1 - s_1),(s_2 - s_1),(e_2 - s_1)) = (0, d_2, d_3, d_4)$$

We then form a set of all these tuples to retain only unique distances. We generate a list of pseudo-reads for unique distances, and compare the result of each list to all observed samples with the same distance tuples. Taking a single tuple of distances (d_1, d_2, d_3, d_4), Chrom-Sig generates *n* pseudo-reads, for which it creates a list of randomly generated values of size *n* using random.randint, and np.random.seed(12345) -- each value x_i in the list follows 0 < x_i < chrom_length – $(e_2 - s_1)$ – 1. It then creates the following list:

$$[[c_1, d_1 + x_1, d_2 + x_1], [c_2, d_3 + x_1, d_4 + x_1], [c_1, d_1 + x_2, d_2 + x_2], [c_2, d_3 + x_2, d_4 + x_2], \ldots, [c_1, d_1 + x_n, d_2 + x_n], [c_2, d_3 + x_n, d_4 + x_n]]$$

This list is created for each unique distance tuple. Each list can then be directly input to pyBedGraph (0.5.43), generating a list of pseudo-enrichment values for each distance tuple. Since reads are paired, every two values in this list are then averaged to find the pseudo-enrichment for each read. The resulting list can then be compared to the observed enrichments of the set of all observed reads with the same distance tuple.

For a single-end read $[c,\ s,\ e]$, this process is much simpler than a paired-end read, since all single-end reads have the same span (*e* – *s*). Rather than forming distance tuples (since they would be the same for all reads), Chrom-Sig simply records the span for all reads $(e - s) = d$. It then generates a list of random values of size *n* using random.randint and np.random.seed(12345), and ensures each value x_i in the list follows 0 < x_i < chrom_length – d – 1 and generates a list:

$$[[c, x_1, x_1 + d], [c, x_2, x_2 + d], \ldots, [c, x_n, x_n + d]]$$

This list is input to pyBedGraph, and the resulting list of pseudo-enrichment values can then be compared to all reads in the current chromosome.

By treating reads with the same span/distance tuple as duplicates, we significantly reduce the amount of data we need to generate, thereby reducing the runtime. For example, for a single-end file with *r* reads for a chromosome and *n* pseudo-samples to produce, rather than having to determine $r * n$ pseudo-enrichments, we only have to determine *n* enrichments for the whole chromosome. Similarly, for a paired-end file, a chromosome with *r* reads might only have a tenth that number of unique spans. Therefore, instead of calculating $r * n$ pseudo-enrichments, we are only calculating $r/10 * n$ enrichments for the chromosome. Another way Chrom-Sig reduces computation time is by calling pyBedGraph as few times as possible. pyBedGraph is a separate library and calling it takes extra time – in paired-end files, instead of determining the enrichment of each pair of fragments in each read at a time, we simply flatten all the fragments for all pseudo reads into a single list. This way, for a single distance, we only need to call pyBedGraph once. Since lists maintain order, we can then recombine enrichments belonging to the same read to form our actual pseudo-enrichment list.

An essential part of the de-noising process is determining the raw p-values of the observed samples based on the null hypothesis that the observed enrichment value is equal to the expected enrichment values; the alternative hypothesis is that the observed enrichment value is greater than the expected. For an observed enrichment *o* and list of expected enrichments $E = [e_1, e_2, \ldots, e_n]$, we calculate the raw p-value as follows:

$$p\text{-val}= \frac{\sum_{i=1}^{n}(o<e_i)}{n}$$

While forming an array $[(o < e_i)]$ and taking the sum of this array may take fewer lines of code, taking the sum of an array is computationally expensive. Instead, Chrom-Sig iterates through each expected enrichment value, comparing it to the observed, and adds the result to a running total to optimize speed. It then divides this total by the number of pseudo samples. Raw p-values for each chromosome are adjusted for multiple hypothesis testing via the Benjamini-Hochberg method with a user pre-defined false discovery rate (FDR) or a significance level α. The reads with adjusted p-value $\leq \alpha$ are considered to be statistically significant and are classified as 'pass'; others are considered insignificant or 'fail'.

Chrom-Sig outputs the 'pass' and 'fail' reads into separate bed files. In order to visualize the results, Chrom-Sig then converts these bed files into bedgraph files. Finally, when each chromosome-wise processes are completed, Chrom-Sig concatenates all the pass beds, fail beds, pass bedgraphs, and fail bedgraphs into "total" files for the whole genome.

Chrom-Sig also produces peak-calling results for both the original input data and the Chrom-Sig results. It runs the SICER2 (1.0.3) algorithm, which takes a bed file and the name of the reference genome as inputs, and outputs a scoreisland file with the locations of the peaks it called. Chrom-Sig runs SICER2 on the original standard bed file, and the total pass-pileup bed file.

For processing single-cell or single-nucleotide data such as snATAC-seq, Chrom-Sig creates the aggregated pseudo-bulk coverage tracks via the method described above, disregarding the fact that each read is derived from a single cell. The statistical significance is computed for each read, retaining the cell barcode annotated in the read ID. Therefore, users can perform downstream single-cell analyses on the reads that are deemed significant by Chrom-Sig.

Supplementary Method S2. `Chrom-Sig` usage and commands

We recommend allocating about an hour of runtime and 25 GB of memory per 50 million paired-end reads.

```
The purpose of this command is to denoise 1-dimensional chromatin profiles. This data must be in single-
end or paired-end format. The command takes a .bam or .bed file as input, and outputs
    denoised .bed, .bedgraph, and .scoreisland files for the full genome (.scoreisland is the result of
the SICER peak-calling algorithm).
    USAGE:
    sbatch multijob_bam2freq_enrich_bg.sh --bamorbed input_file --dir data_directory --r ref --fdr fdr -
-num samp_size --type str_type --cov coverage --lib lib_name --plot true --modload true

        ARGUMENTS:
            --bamorbed      Input bam or bed file containing your dataset.
            --dir           Directory containing the bam/bed file, as well as your reference genome
sizes file (ex: /Users/chromsig/Data/).
            --r             Name of reference genome (ex: hg38).
            --fdr           FDR threshold for denoising (ex: 0.1).
            --num           Number of pseudo samples to be generated for denoising.
            --type          Type of read in your dataset, Single End or Paired End (ex: SE or PE).
            --cov           Minimum read coverage required for a chromsome to be denoised (ex.: To set
minimum coverage for a chromsome to be 5%, enter --cov 0.05)

        OPTIONS:
            --lib           Name of dataset library (ex: For GM12878_ATAC-seq_ENCFF415FEC.bam, this
would be ENCFF415FEC).
            --plot          Produce histogram plots of distances between read fragments (only for Paired
End), type 'true' if you want plots, skip this option if you do not.
            --modload       Load python 3.9, bedtools, and samtools; type 'true' if you want to use
module load to load these, skip this option if you do not.

        EXAMPLE:
            sbatch multiscript_bam2freq_enrich_bg.sh --bamorbed GM12878_ATAC-seq_ENCFF415FEC.bam --dir
/nfs/turbo/umms-minjilab/njgupta/chromsig/ATAC-seq_PE/ --r hg38 --fdr 0.1 --num 5000 --type PE --cov
0.05"
```

Supplementary Method S3. Analysis of `Chrom-Sig` results

CTCF Motifs

To assess the precision of CTCF binding site identification before and after Chrom-Sig with FDR 0.1 and 5000 pseudo-reads, we implemented a python script using python 3.11.11 with matplotlib version 3.9.2, pybedtools 0.10.0, and numpy 2.2.1. The analysis looks at GM12878 CTCF ChIP-seq ENCFF355CYX (36,269 peaks original, 24,872 peaks after Chrom-Sig) as well as GM12878 CTCF CUT&RUN replicates 4DNFI2G71DR4 (55,251 peaks original, 22,554 peaks after Chrom-Sig) and 4DNFI9U71IB4 (62,176 peaks original, 19,233 peaks after Chrom-Sig).

CTCF motif locations were provided in bed format called by STORM as described in Tang et al., 2015. Peak files were provided in a bed format. Genomic intervals were loaded using pybedtools and the number of regions in each file were counted. We defined precision as the overlap of peaks with CTCF motifs divided by the number of total peaks. Overlap between CTCF motifs and peak regions was calculated using pybedtools intersect with parameter u=True to obtain the number of CTCF motif regions in each peak file. Both the Chrom-Sig pass pileup SICER peak file and the SICER peak calls on the original data were analyzed for each sample.

MEME Analysis

In continuity with the above analysis, motif analysis was performed on GM12878 CTCF CUT&RUN 4DNFI2G71DR4 for the original data and after Chrom-Sig with FDR 0.1 and 5000 pseudo-reads. The MEME suite (v5.5.5) was used to identify enriched DNA sequence motifs within peak regions using the command line version. The input required for this process is peak region file in a bed format, a reference genome, and motif database. We used bedtools version 2.31.1 to convert to fasta through the getfasta command with hg38 reference genome from iGenomes/Homo_sapiens/UCSC/hg38/Sequence/BWAIndex/genome.fa. The motif database HUMAN/HOCOMOCOv11_core_HUMAN_mono_meme_format.meme was downloaded from MEME. The meme-chip tool was run with default parameters (-time 240 -ccut 100 -dna -order 2 -minw 6 -maxw 15 -db HUMAN/HOCOMOCOv11_core_HUMAN_mono_meme_format.meme -meme-mod zoops -meme-nmotifs 5 -meme-searchsize 100000 -streme-pvt 0.05 -streme-align center -streme-totallength 4000000 -centrimo-score 5.0 -centrimo-ethresh 10.0) on each fasta file. The output was an html meme report with motifs, E-value, discovery program, and known motif matches from the database. We reported at the top 3 motifs for both before ('original') and after Chrom-Sig ('pass'). The script "run_meme.sh" was run on a cluster with 10G of memory and took less than an hour to run for each peak file.

ChromHMM States

This analysis compares the distribution of ChromHMM states in original and Chrom-Sig processed datasets across GM12878 ENCFF646NWY ATAC-seq data and K562 RNAPII ChIP-seq replicates ENCFF480AJZ and ENCFF785OCU. ATAC-seq peaks and RNAPII ChIP-seq peaks are known to be correlated with promotors and enhancers and our aim was to evaluate whether Chrom-Sig enhances such known correlations.

A script named "chromHMM_annotation.sh" took as input previously annotated region files for GM12878 and K562 cell lines and the aforementioned peak files for both original data and Chrom-Sig. The script used bedtools 2.31.1 intersect with option -wao to get the size of the intersection where -a is the peak file and -b is the annotation file. The output was saved as the peak file name with "_chromhmmanno.bed" extension.

A custom python workflow named "chromsig_chromhmm_annotation.ipynb" takes the annotated files as input and outputs the plots in the manuscript. The method uses Python 3.9.23 and packages numpy and matplotlib. ChromHMM states are grouped to reduce the state space from 15 to 7. The 7 groups are promoter, enhancer, transcription, insulator, repressed polycomb, heterochromatin, or other. The percentage of each state is calculated by summing the number of intersecting base pairs for each state and dividing by the total base pairs. These percentages were calculated for both original and Chrom-Sig processed datasets and are shown as stacked bar charts.

AtacWorks

To benchmark Chrom-Sig against existing methods, we compared its performance to AtacWorks, a tool which can be used for denoising 1-D genomic signal tracks. Input data consisted of bedgraph files for two GM12878 CTCF ChIP-seq replicate pairs, one GM12878 CTCF CUT&RUN replicate pair, and one GM12878 ATAC-seq replicate pair. These files were first converted to the required bigwig format using the bedGraphToBigWig utility from UCSC. Following the AtacWorks tutorial, we processed the bigwig files using the AtacWorks denoise command with its pre-trained "Bulk ATAC-Seq low quality 20M reads" model and default parameters. The resulting denoised tracks were post-processed using the peak_summary script to format peaks and filter out those shorter than 20 bp. All AtacWorks jobs were run on a system equipped with two 2.4 GHz Intel Xeon Gold 6148 CPUs and two NVIDIA Tesla V100 16GB GPUs. For a direct comparison, the final peak bed files

generated by AtacWorks were evaluated using the same metrics applied to the Chrom-Sig output. Specifically, CTCF experiment outputs were assessed for CTCF motif precision and via MEME analysis, while ATAC-seq outputs were evaluated for enrichment in relevant chromHMM states, as detailed in the above section.

Supplementary Table S1. Details of 19 datasets used in the benchmark

BAM File ID	Cell line	Assay type	Factor	Read type	# of uniquely mapped reads
ENCFF453EJM	GM12878	ChIP-seq	CTCF	36 bp SE	15,574,449
ENCFF355CYX	GM12878	ChIP-seq	CTCF	36 bp SE	7,508,508
ENCFF500UFQ	GM12878	ChIP-seq	CTCF	33 bp SE	10,020,218
ENCFF668ROZ	GM12878	ChIP-seq	CTCF	33 bp SE	6,119,969
ENCFF481OXC	GM12878	ChIP-seq	YY1	36 bp SE	16,919,080
ENCFF676OVC	GM12878	ChIP-seq	YY1	36 bp SE	14,333,464
ENCFF493HLV	GM12878	ChIP-seq	YY1	28 bp SE	4,443,118
ENCFF301QON	GM12878	ChIP-seq	YY1	28 bp SE	6,900,603
ENCFF431RHO	GM12878	ChIP-seq	RAD51	100 bp PE	42,035,179
ENCFF725MWO	GM12878	ChIP-seq	RAD51	100 bp PE	20,626,767
ENCFF480AJZ	K562	ChIP-seq	POLR2A	100 bp PE	20,026,319
ENCFF785OCU	K562	ChIP-seq	POLR2A	100 bp PE	25,722,407
ENCFF415FEC	GM12878	ATAC-seq		101 bp PE	23,478,485
ENCFF646NWY	GM12878	ATAC-seq		101 bp PE	24,897,361
ENCFF512VEZ	K562	ATAC-seq		101 bp PE	25,799,449
ENCFF987XOV	K562	ATAC-seq		101 bp PE	23,893,400
4DNFI2G71DR4	GM12878	CUT&RUN	CTCF	25 bp PE	7,454,808
4DNFI9U71IB4	GM12878	CUT&RUN	CTCF	25 bp PE	9,756,208
ENCFF995ATT	GM12878	snATAC-seq		50 bp PE	174,483,824

The 17 ENCODE and 2 4D Nucleome datasets used in the benchmark. BAM File ID: unique identification assigned by ENCODE/4D Nucleome; Cell line: name of the cell line listed by ENCODE/4DN; Assay type: experimental assay; Factor: protein immunoprecipitation factor if ChIP-seq; Read type: read length and SE (single-end) or PE (paired-end); # of uniquely mapped reads: read counts in the BAM file.

Supplementary Table S2. Runtime, memory, statistics for 5000 pseudo-reads

Dataset	# uniq. Reads (millions)	FDR 0.2 Runtime (s)	FDR 0.2 Memory (GB)	FDR 0.2 Pass # (mil)	FDR 0.2 Fail # (mil)	FDR 0.1 Runtime (s)	FDR 0.1 Memory (GB)	FDR 0.1 Pass # (mil)	FDR 0.1 Fail # (mil)
GM12878_CTCF_ChIP-seq_ ENCFF453EJM	15.57	749.37	4.5	3.10	12.47	712.94	4.5	1.90	13.67
GM12878_CTCF_ChIP-seq_ ENCFF355CYX	7.51	480.14	2.2	1.73	5.78	332.24	2.4	1.11	6.40
GM12878_CTCF_ChIP-seq_ ENCFF500UFQ	10.02	909.96	3.0	3.16	6.86	499.58	3.0	2.76	7.26
GM12878_CTCF_ChIP-seq_ ENCFF668ROZ	6.12	300.04	1.7	2.15	3.97	298.77	2.0	1.89	4.23
GM12878_YY1_ChIP-seq_ ENCFF481OXC	16.92	811.26	4.5	3.57	13.35	858.67	4.5	2.34	14.58
GM12878_YY1_ChIP-seq_ ENCFF676OVC	14.33	715.52	4.0	3.67	10.66	697.67	3.6	2.52	11.81
GM12878_YY1_ChIP-seq_ ENCFF493HLV	4.44	217.16	1.9	0.06	4.38	494	1.9	0.02	4.42
GM12878_YY1_ChIP-seq_ ENCFF301QON	6.90	448.27	2.5	0.11	6.79	562.97	2.5	0.04	6.86
GM12878_ChIP-seq_RAD51_ ENCFF431RHO	42.04	2562.56	19	5.97	36.07	3058.42	19	2.85	39.19
GM12878_ChIP-seq_RAD51_ ENCFF725MWO	20.63	3739.58	11	2.39	18.24	3819.52	11	1.04	19.59
K562_ChIP-seq_POLR2A_ ENCFF480AJZ	20.03	2248.78	9.7	7.94	12.09	2302.57	12	4.84	15.19
K562_ChIP-seq_POLR2A_ ENCFF785OCU	25.72	2886.76	14	13.02	12.7	2669.15	12	8.52	17.2
GM12878_ATAC-seq_ ENCFF415FEC	23.48	8206.45	15	17.90	5.58	7918.71	14	15.74	7.74
GM12878_ATAC-seq_ ENCFF646NWY	24.90	6326.74	17	17.34	7.56	9139.48	15	14.62	10.28
K562_ATAC-seq_ ENCFF512VEZ	25.80	5219.7	19	14.93	10.87	5642.89	19	11.84	13.96
K562_ATAC-seq_ ENCFF987XOV	23.89	5974.06	17	13.12	10.77	5089.13	18	10.09	13.8
GM1878_CUTandRUN_CTCF_ 4DNFI2G71DR4	7.45	5345.52	3.9	2.36	5.09	5283.35	3.9	1.53	5.92
GM1878_CUTandRUN_CTCF_ 4DNFI9U71IB4	9.76	4172.32	4.9	3.20	6.56	4621.53	4.5	2.21	7.55
GM12878_snATAC-seq_ ENCFF995ATT	174.48	15510.1	83	129.55	44.93	11279	85	111.78	62.7
AVERAGE	25.26	3517	12.52	12.9	12.35	3435.8	12.52	10.4	14.86

All 19 datasets with the number of uniquely mapped reads, followed by the runtime, memory usage, and the number of pass and fail reads after running Chrom-Sig using false discovery rate (FDR) of 0.2 and 0.1.

Supplementary Figure S1. Overview of `Chrom-Sig`

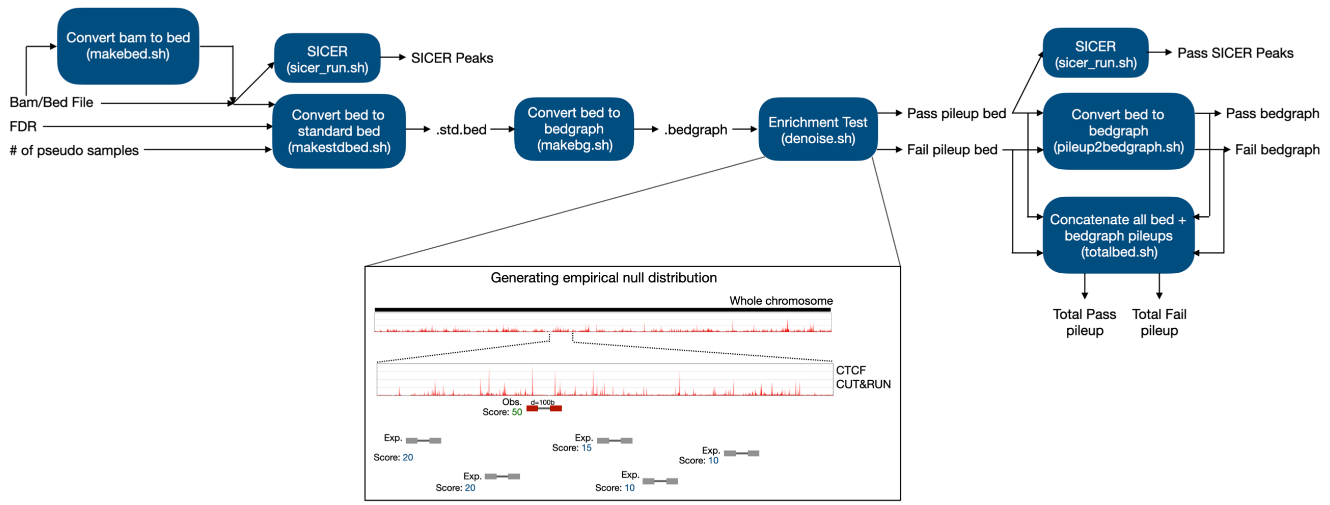

Chrom-Sig pipeline. If taking a BAM file as input, converts it to a bed file, otherwise takes a bed file as input. FDR: False Discovery Rate threshold. # of pseudo samples: the number of pseudo-reads for the Enrichment Test (denoise.sh) to generate. The pipeline converts a bed file to a standard bed file by sorting the data and removing non-standard chromosomes. The standard bed file is converted to a bedgraph, which is then input along with the bed file to the Enrichment Test. This test generates a number of pseudo-reads specified by the user, determines the maximum value in the bedgraph along each of those pseudo-reads (for paired end data, taking the average of the maximums along each fragment), and compares the maximum along the observed read (from the bed file) to the pseudo-reads to generate a p-value. If the p-value is below the FDR threshold, the read passes and is copied to a pass-pileup bed file, otherwise it is copied to a fail-pileup bed file. Chrom-Sig then coverts the pass and fail bed files for each chromosome to bedgraph files, and concatenates each set of files into a total file for the whole genome. The SICER peak-calling algorithm is then run on the initial bed file and the total pass-pileup bed file.

Supplementary Figure S2. Overview of Datasets

a

b

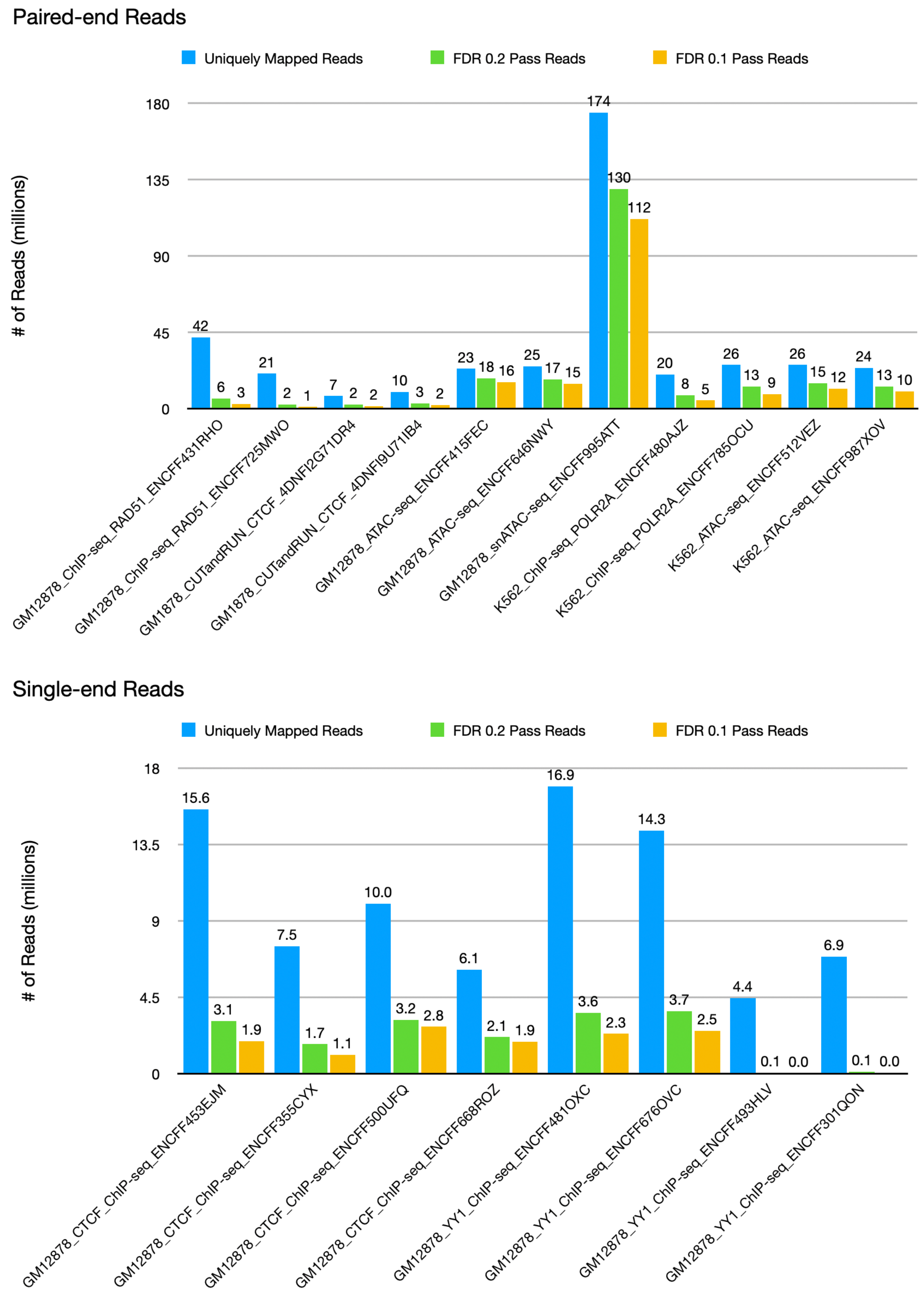

Uniquely mapped reads, number of reads retained for FDR threshold of 0.1, and number of reads retained for FDR threshold of 0.2. Uniquely mapped reads were obtained from the original bed file for each dataset. The number of reads is counted in millions. a) 11 paired-end datasets. b) 8 single-end datasets.

Supplementary Figure S3. Runtime and Memory

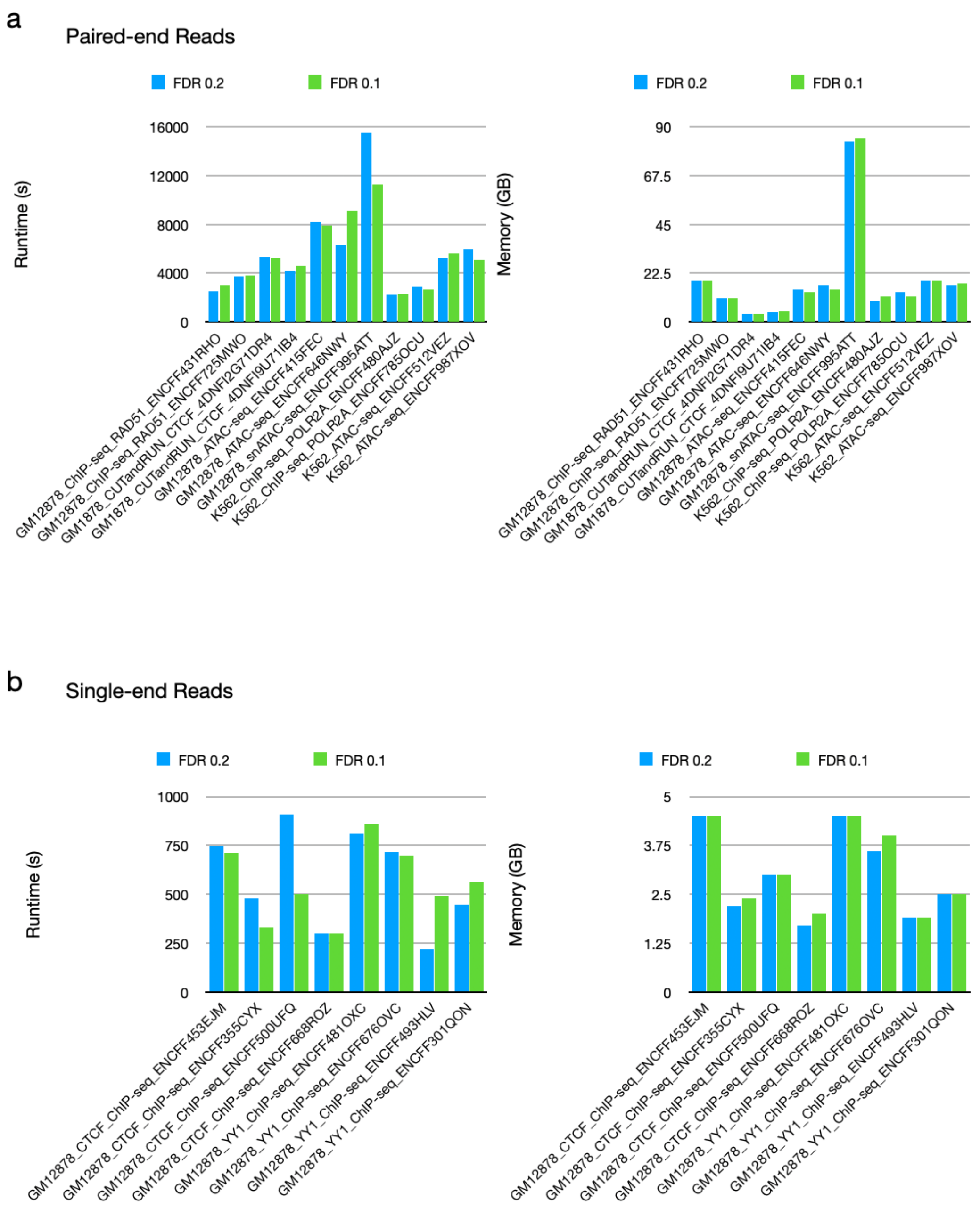

Runtime and memory usage for FDR thresholds of 0.2 and 0.1. All runs were done with 5000 samples. Runtime is in seconds, and memory usage is in gigabytes. a) paired-end reads data, b) single-end reads data.

Supplementary Figure S4. Runtime and Pass % vs. Number of Pseudo-Reads

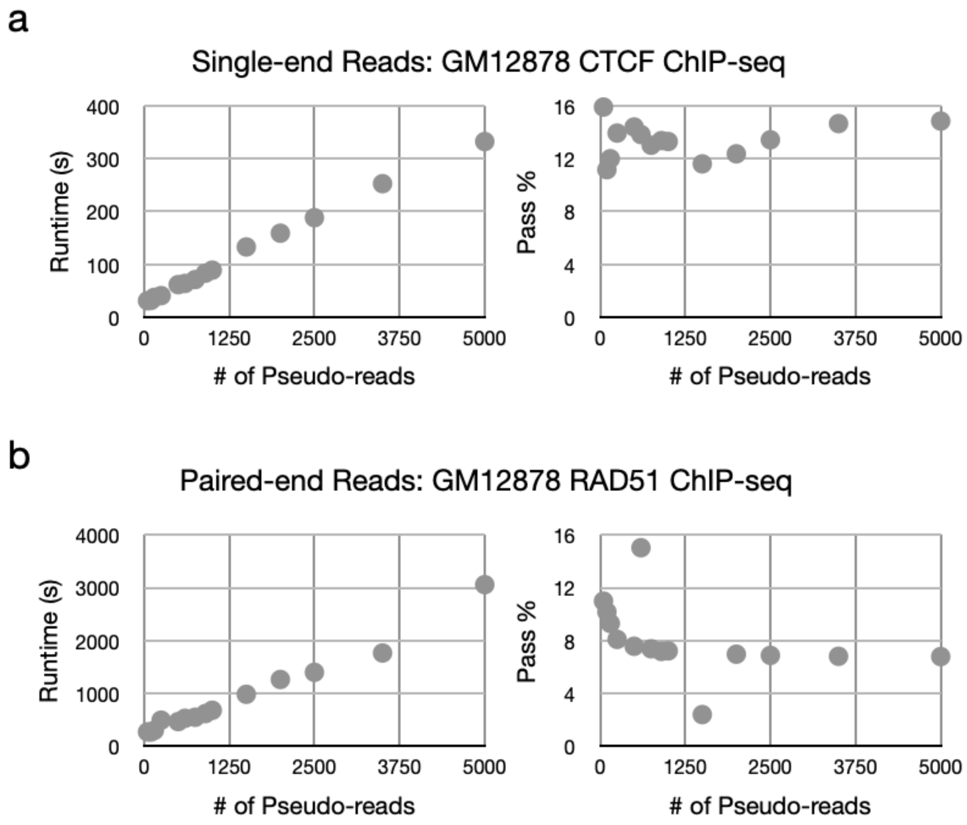

For one single-end (panel a) and one paired-end (panel b) dataset, given pseudo-reads from 50 to 5000, the runtime and pass percentage for each run. Given pseudo-reads were 50, 100, 150, 250, 500, 600, 750, 900, 1000, 1500, 2000, 2500, 2500, and 5000. All runs were done with FDR 0.1.

Supplementary Figure S5. `Chrom-Sig` fail output vs. IgG control

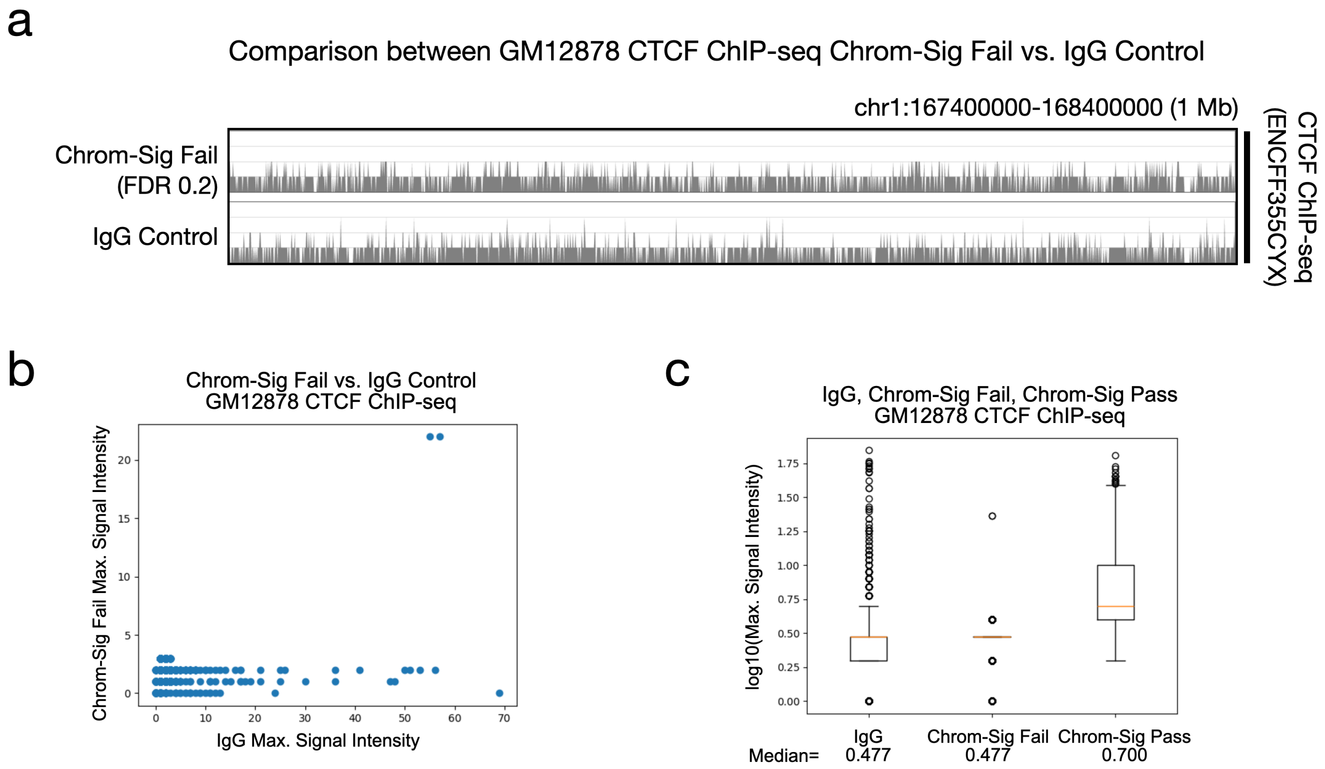

Comparison between Chrom-Sig fail output and IgG control file. Chrom-Sig was run on a Single-End GM12878 CTCF ChIP-seq file with ID ENCFF355CYX, with parameters FDR 0.2, and 5000 pseudo-reads generated. a) Bedgraph visualization of Chrom-Sig fail pileup, and bedgraph visualization of IgG control. b) Scatterplot comparing maximum bedgraph values along 5 kb intervals genome-wide for Chrom-Sig fail output and IgG control file. c) Boxplot showing distribution of maximum bedgraph values along 5 kb intervals genome-wide for IgG control file, Chrom-Sig fail output, and Chrom-Sig pass output; signal intensity along y-axis is scaled by log10 of maximum signal intensity values.

Supplementary Figure S6. Paired-end Reads Example for GM12878

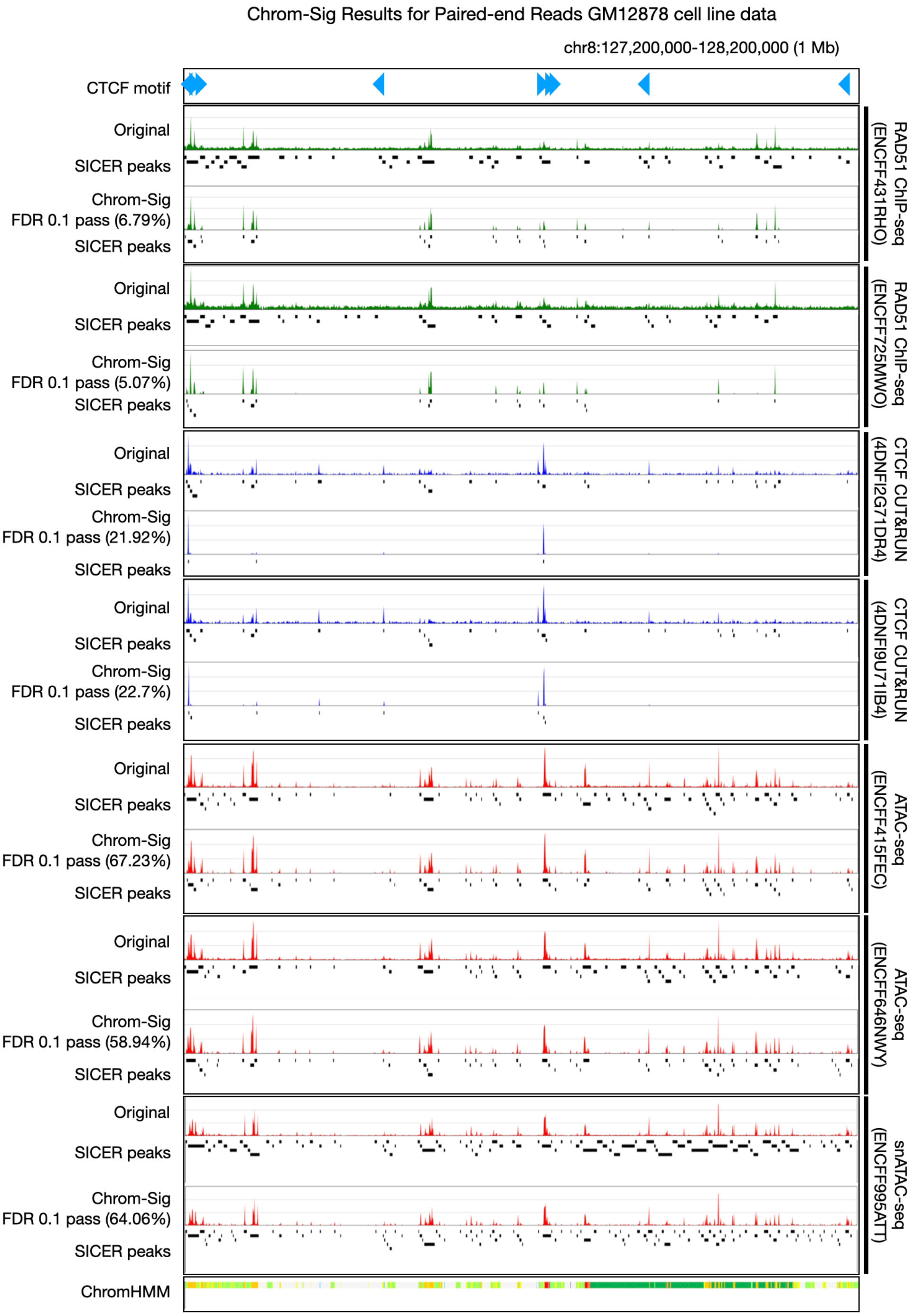

Chrom-Sig results for all paired-end datasets from GM12878 cell-line, visualized in the genome browser. CTCF Motif: CTCF binding sites with orientation. Original: Bedgraph file generated directly from input BAM/bed file. SICER peaks: Bed file result of running SICER algorithm on the original bedgraph file. Chrom-Sig FDR 0.1 pass: pass bedgraph generated from original bedgraph by Chrom-Sig (percentage refers to how many reads were retained by Chrom-Sig result from original bedgraph). SICER peaks (below Chrom-Sig FDR 0.1 pass): Bed file from SICER algorithm run on pass-pileup bed generated by Chrom-Sig. ChromHMM: Chromatin states.

Supplementary Figure S7. Paired-end Reads Example for K562

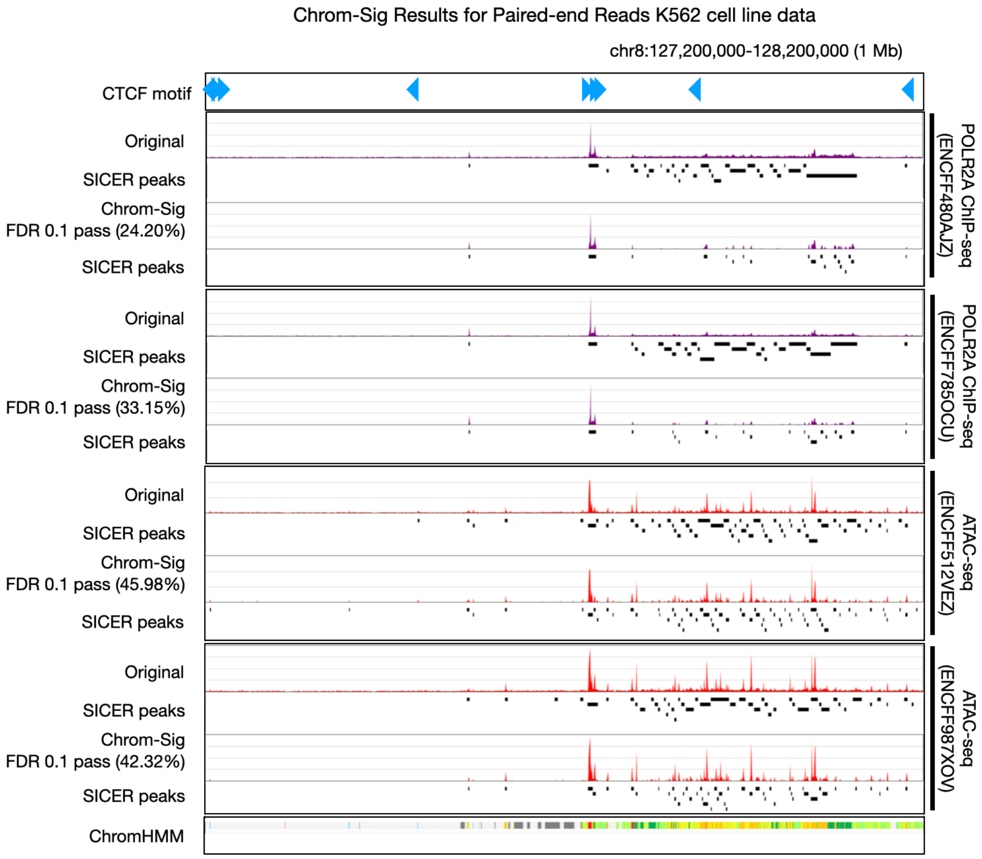

Chrom-Sig results for all paired-end datasets from K562 cell-line, visualized in the genome browser. CTCF Motif: CTCF binding sites with orientation. Original: Bedgraph file generated directly from input BAM/bed file. SICER peaks: Bed file result of running SICER algorithm on the original bedgraph file. Chrom-Sig FDR 0.1 pass: pass bedgraph generated from original bedgraph by Chrom-Sig (percentage refers to how many reads were retained by Chrom-Sig result from original bedgraph). SICER peaks (below Chrom-Sig FDR 0.1 pass): Bed file from SICER algorithm run on pass-pileup bed generated by Chrom-Sig. ChromHMM: Chromatin states.

Supplementary Figure S8. Single-end Reads Example

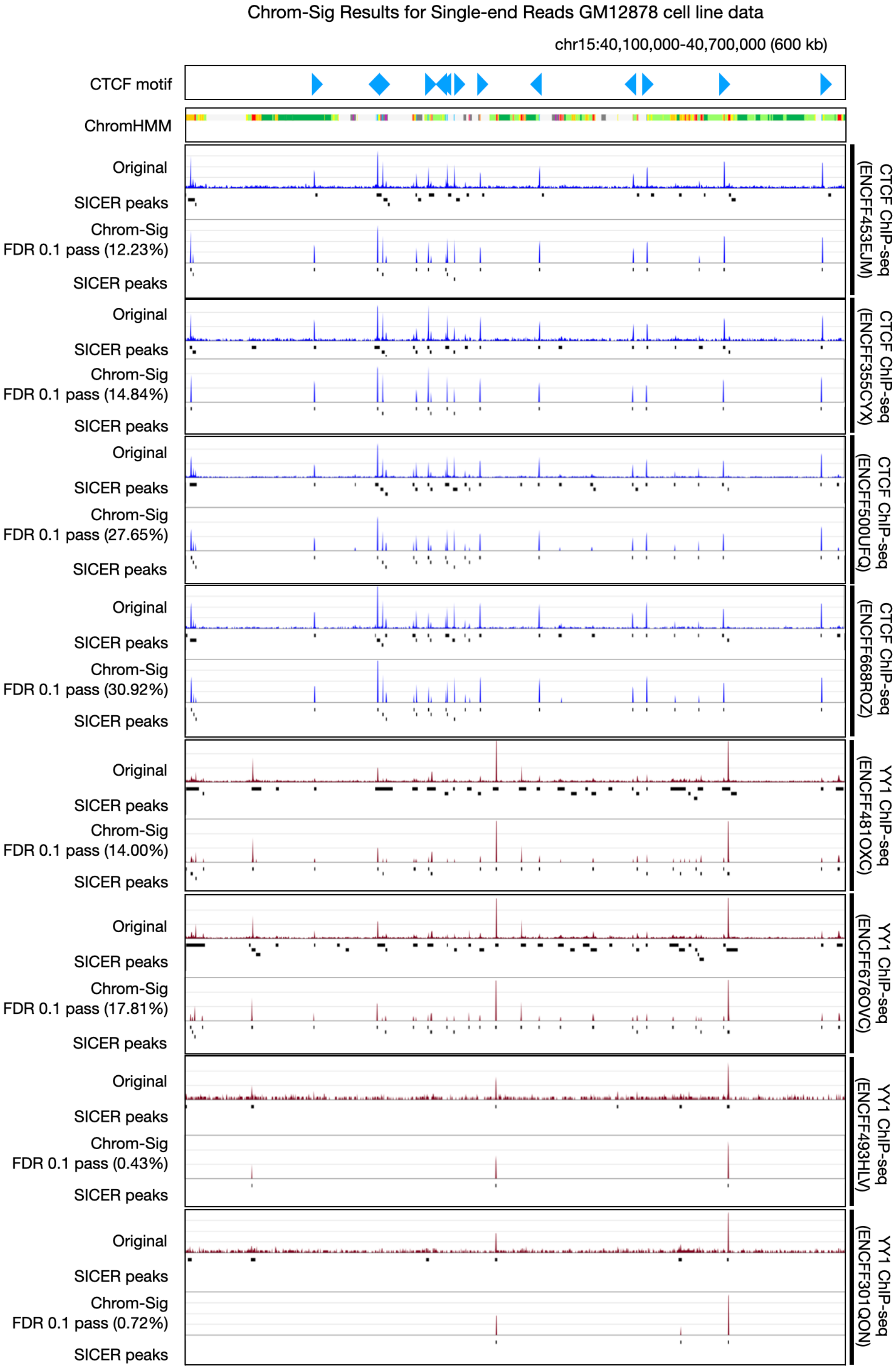

Chrom-Sig results for all single-end datasets (all single-end data is from GM12878 cell-line), visualized in the genome browser. CTCF Motif: CTCF binding sites with orientation. Original: Bedgraph file generated directly from input BAM/bed file. SICER peaks: Bed file result of running SICER algorithm on the original bedgraph file. Chrom-Sig FDR 0.1 pass: pass bedgraph generated from original bedgraph by Chrom-Sig (percentage refers to how many reads were retained by Chrom-Sig result from original bedgraph). SICER peaks (below Chrom-Sig FDR 0.1 pass): Bed file from SICER algorithm run on pass-pileup bed generated by Chrom-Sig. ChromHMM: Chromatin states.

Supplementary Figure S9. SICER Peaks in Paired-end Reads Data Before and After `Chrom-Sig`

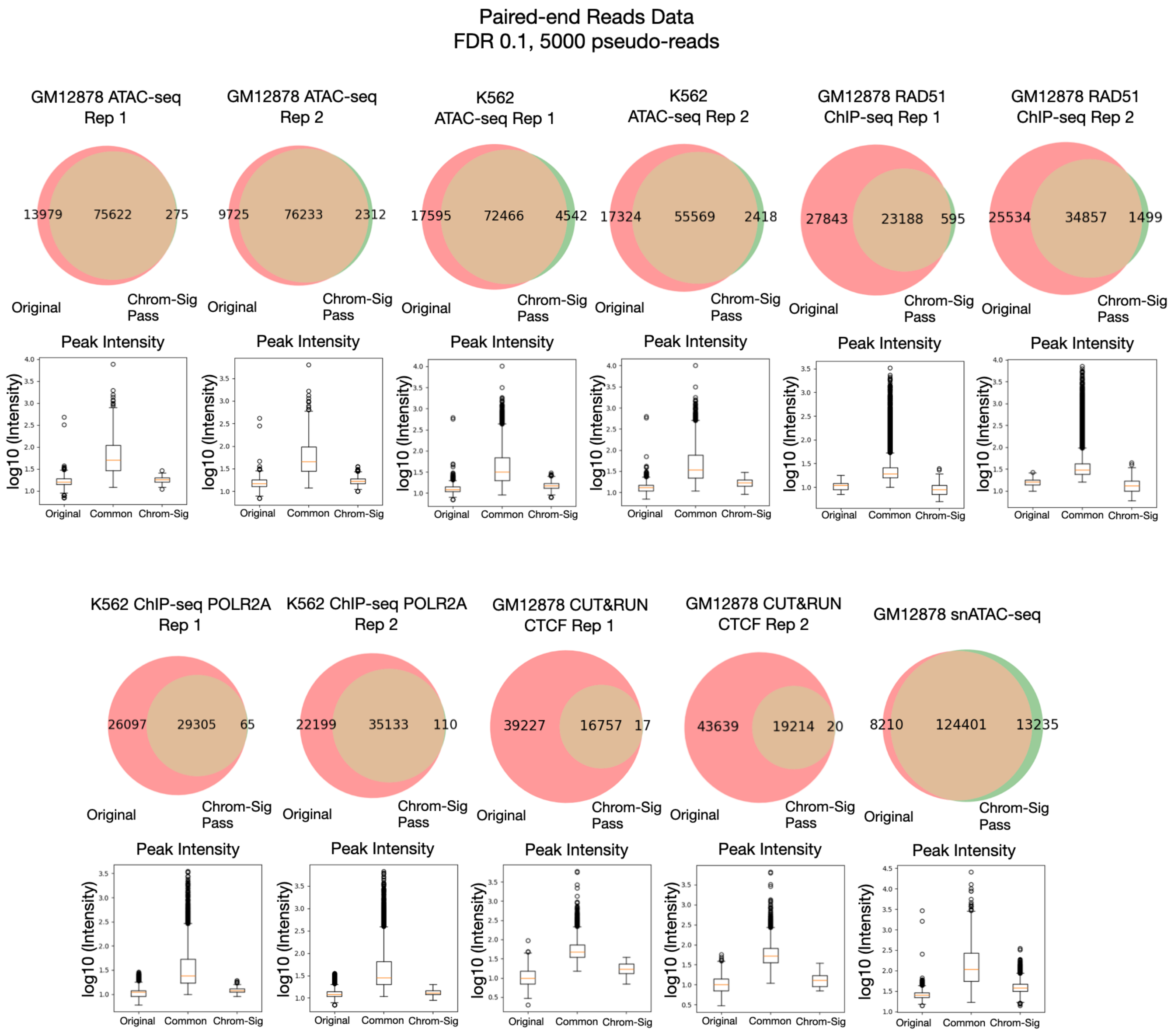

Peaks called by SICER before and after running Chrom-Sig on all paired-end datasets.
Venn Diagram: Original: number of peaks called by SICER when run on bed file input (or directly generated from input).
Chrom-Sig pass: number of peaks called by SICER on total (whole genome) pass-pileup bed file generated by Chrom-Sig.
Box Plot: Maximum intensity of peak locations found by SICER. Intensity is determined by locating the peaks in the corresponding bedgraph file and determining the maximum using pyBedGraph. Original: Intensities of only Original SICER peaks from original bedgraph file. Chrom-Sig: Intensities of only pass-pileup SICER peaks from pass-pileup bedgraph file. Common: Intensities of SICER peaks located in both original and pass-pileup bed files. Intensity is plotted on a log10 scale.

Supplementary Figure S10. SICER Peaks in Single-end Reads Data Before and After `Chrom-Sig`

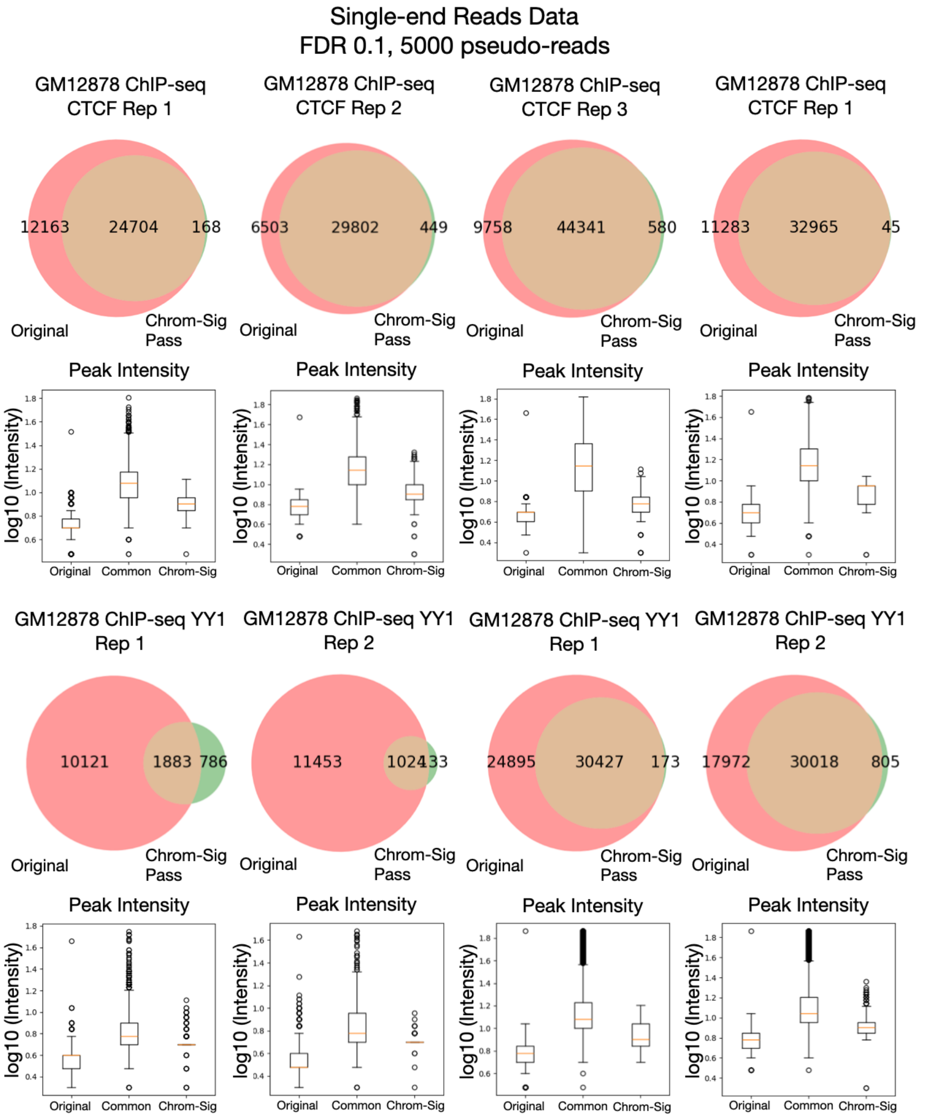

Peaks called by SICER before and after running Chrom-Sig on all single-end datasets.
Venn Diagram: Original: number of peaks called by SICER when run on bed file input (or directly generated from input). Chrom-Sig pass: number of peaks called by SICER on total (whole genome) pass-pileup bed file generated by Chrom-Sig. Box Plot: Maximum intensity of peak locations found by SICER. Intensity is determined by locating the peaks in the corresponding bedgraph file and determining the maximum using pyBedGraph. Original: Intensities of only Original SICER peaks from original bedgraph file. Chrom-Sig: Intensities of only pass-pileup SICER peaks from pass-pileup bedgraph file. Common: Intensities of SICER peaks located in both original and pass-pileup bed files. Intensity is plotted on a log10 scale.

Supplementary Figure S11. CTCF Motif Analyses

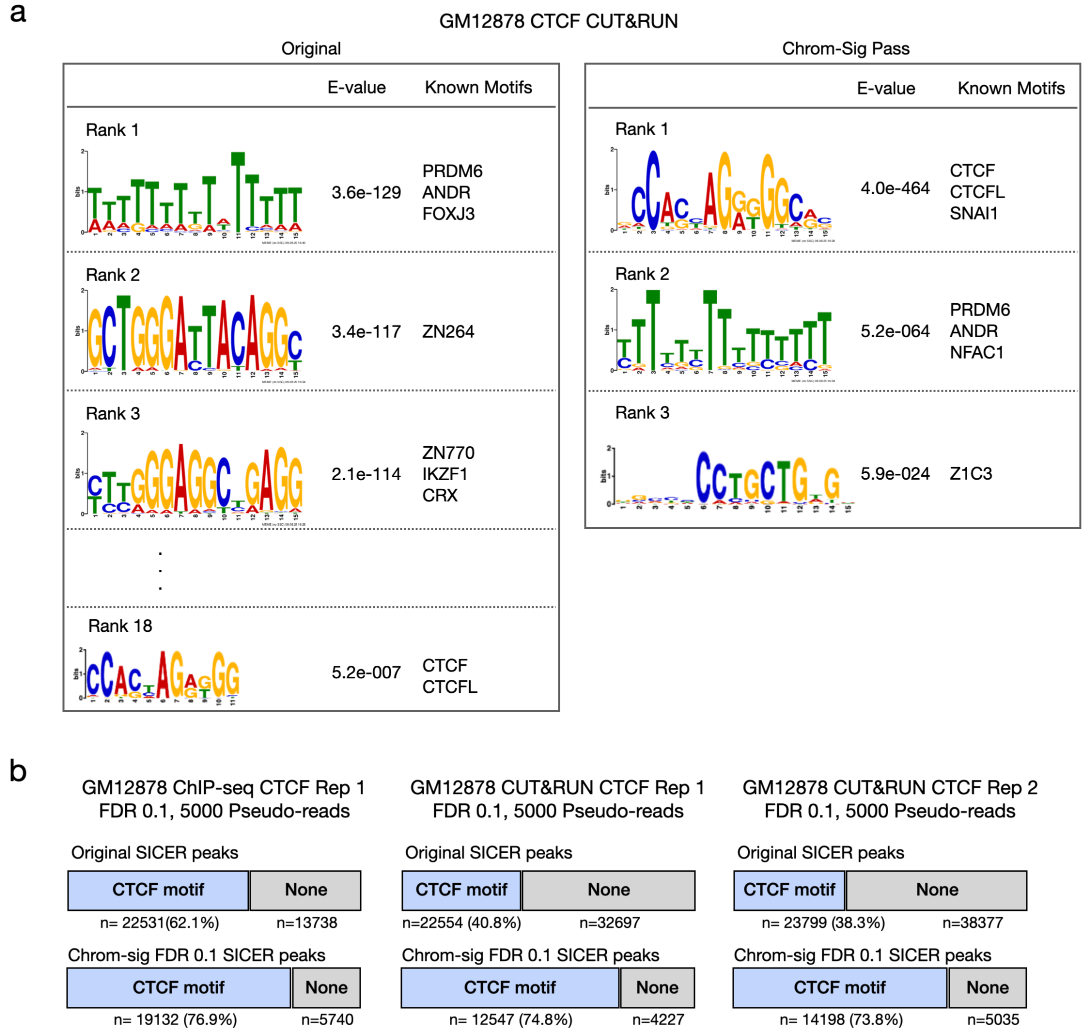

a) Top enriched motifs, E-value, and matching motifs from MEME-Chip for GM12878 CUT&RUN CTCF 4DNFI2G71DR4 before and after Chrom-Sig. b) Comparison of CTCF motif precision between original data and Chrom-Sig with FDR 0.1 and 5000 pseudo-reads for GM12878 ChIP-seq CTCF ENCFF355CYX, GM12878 CUT&RUN CTCF 4DNFI2G71DR4 and 4DNFI9U71IB4.

Supplementary Figure S12. ChromHMM State Annotation Distribution

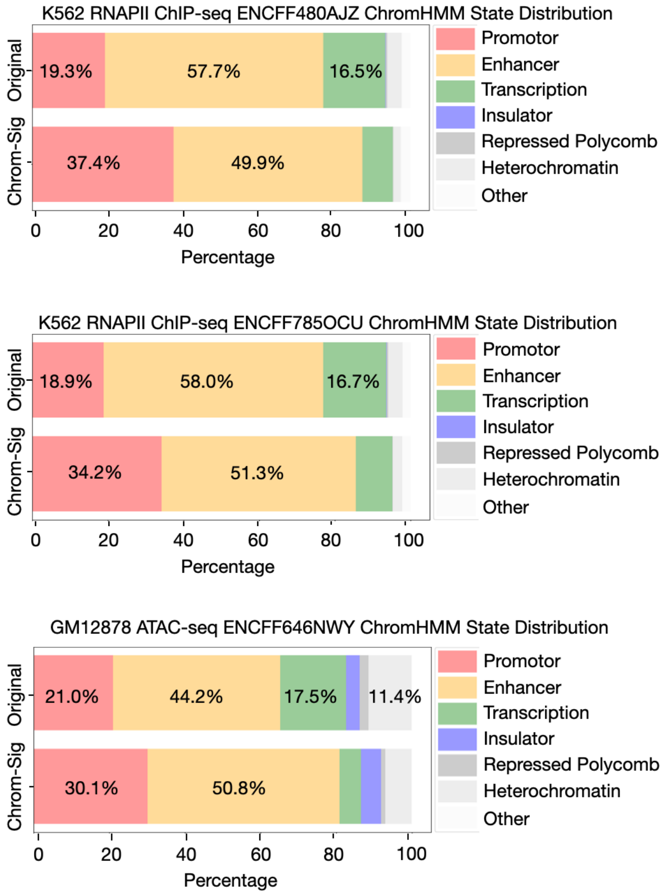

Comparison of the distribution of chromHMM states between original data and Chrom-Sig with FDR 0.1 and 5000 pseudo-reads for K562 RNAPII ChIP-seq ENCFF480AJZ and ENCFF785OCU and GM12878 ATAC-seq ENCFF646NWY. The proportion of enhancer and promotor states increases when Chrom-Sig is applied to the data. Between K562 RNAPII ChIP-seq replicates there is an average of 12.3% higher distribution of enhancers and promotors (ENCFF480AJZ: 77% original vs 87.3% Chrom-Sig and ENCFF785OCU: 76.9% original vs 85.5% Chrom-Sig). In ATAC-seq data, the percentage of transcription and heterochromatin states drops from 28.9% to 12.6% after Chrom-Sig.

Supplementary Figure S13. `Chrom-Sig` vs. AtacWorks

a

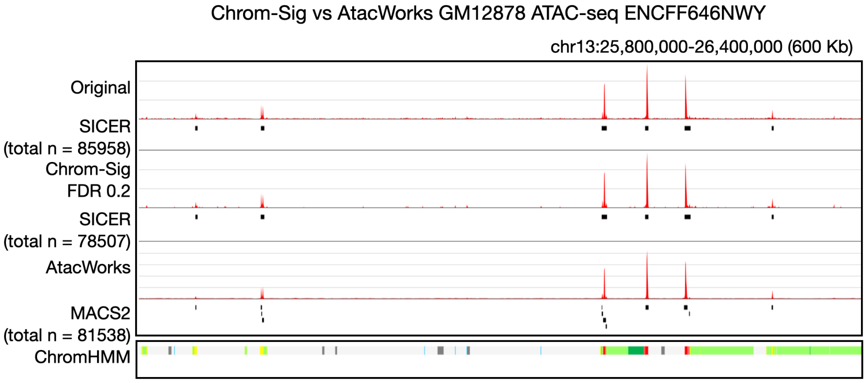

b

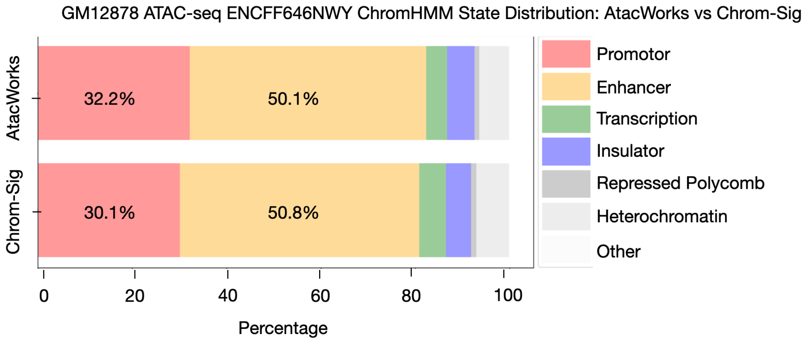

Comparison of Chrom-Sig de-noised bedgraph and peaks vs. AtacWorks de-noised bedgraph and peaks run on GM12878 ATAC-seq Rep 2 (ENCFF646NWY) dataset. a) Genome browser visualization. From top to bottom: original bedgraph file, SICER peak calling run on original file (85,958 total peaks), Chrom-Sig de-noised output run with FDR 0.2 and 5000 pseudo-reads, SICER run on Chrom-Sig output (78,507 peaks), AtacWorks de-noised output, MACS2 peak calling run on AtacWorks output (81,538 peaks), and ChromHMM (Chromatin states). b) Comparison of the distribution of chromHMM chromatin states on peaks called by AtacWorks (top) and Chrom-Sig (bottom).

Supplementary Figure S14. `Chrom-Sig` vs. AtacWorks CUT&RUN

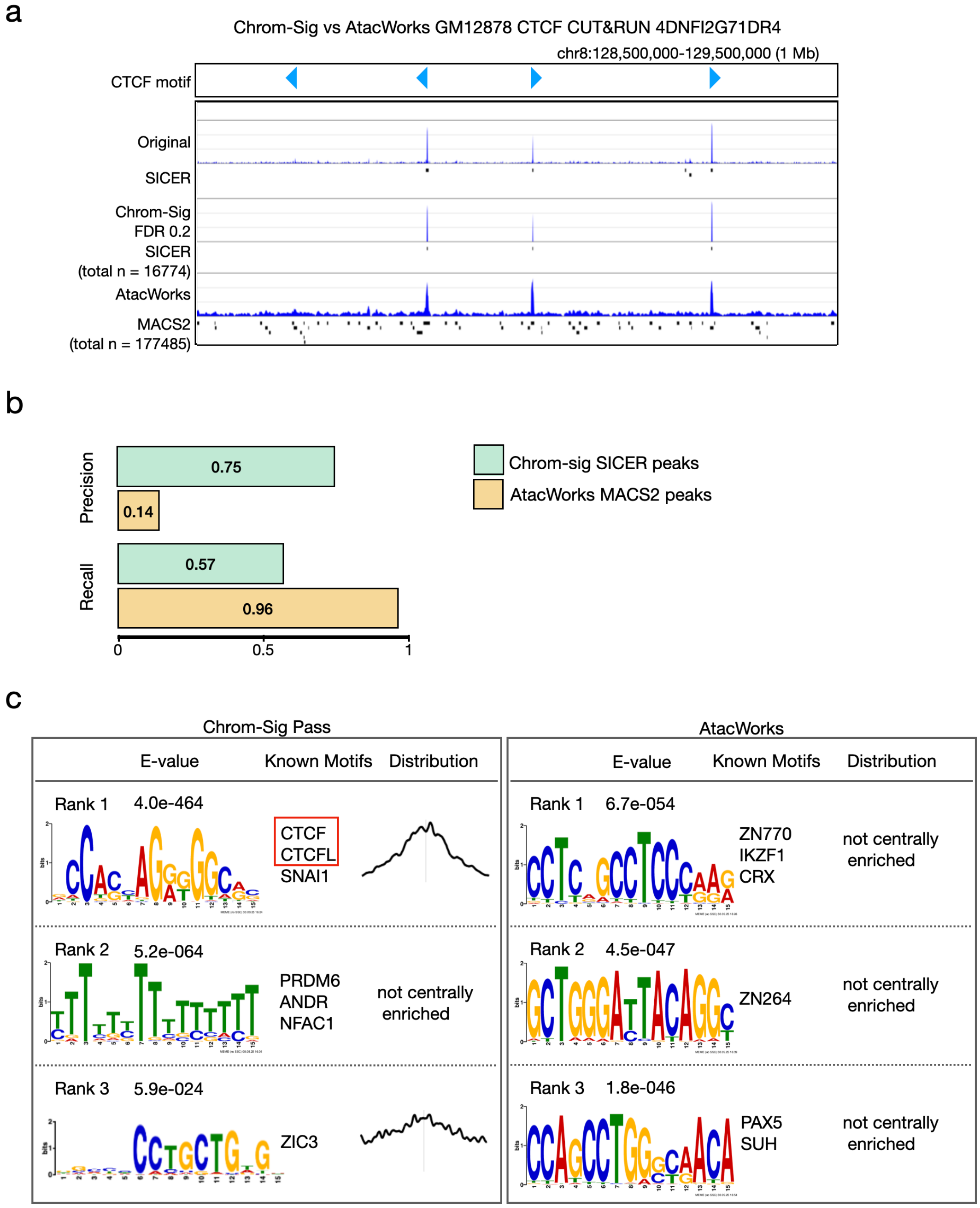

Analysis of Chrom-Sig vs. AtacWorks de-noising run on GM12878 CTCF CUT&RUN Rep 2 (4DNFI2G71DR4) dataset. a) Genome browser visualization of (top to bottom): CTCF Motif: CTCF binding sites with orientation; Original bedgraph file; SICER peak-calling run on original bedgraph file; Chrom-Sig de-noised bedgraph from running on original file with FDR 0.2 and 5000 pseudo-reads; SICER run on Chrom-Sig output (16,774 total peaks in SICER file); AtacWorks de-noised bedgraph from running on original file; MACS2 peak-calling run on AtacWorks output (177,485 total peaks in MACS2 file). b) Precision and recall comparison of CTCF overlap between Chrom-Sig SICER peaks and AtacWorks MACS2 peaks, with Chrom-Sig result in green and AtacWorks result in yellow. c) Top enriched motifs, E-value, and matching motifs from MEME-Chip for GM12878 CUT&RUN CTCF 4DNFI2G71DR4 Chrom-Sig and AtacWorks.

Supplementary Figure S15. `Chrom-Sig` vs. AtacWorks ChIP-seq

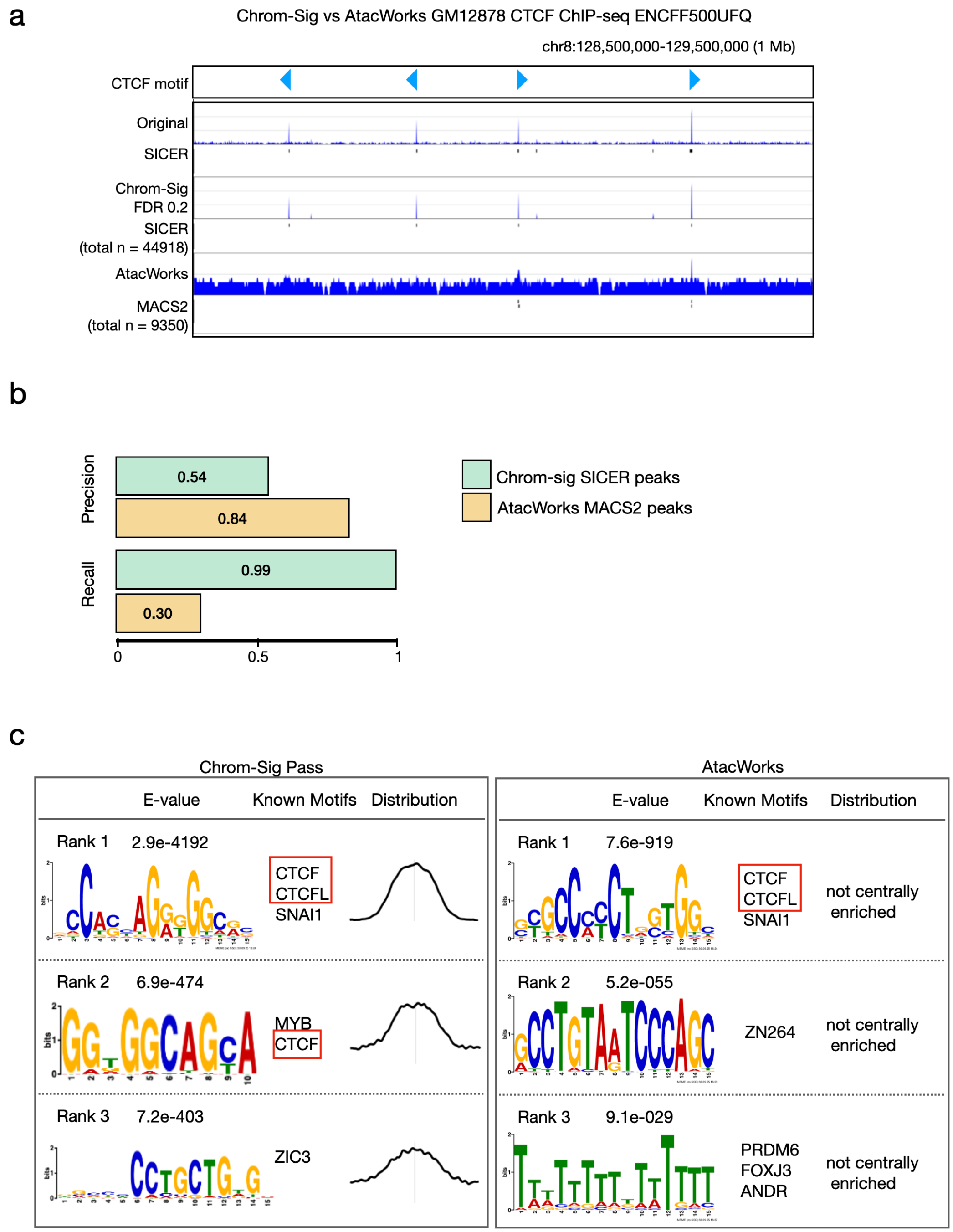

Analysis of Chrom-Sig vs. AtacWorks de-noising run on GM12878 CTCF ChIP-seq (ENCFF500UFQ) dataset. a) Genome browser visualization of (top to bottom): CTCF Motif: CTCF binding sites with orientation; Original bedgraph file; SICER peak calling run on original bedgraph file; Chrom-Sig de-noised bedgraph from running on original file with FDR 0.2 and 5000 pseudo-reads; SICER run on Chrom-Sig output (44,918 total peaks in SICER file); AtacWorks de-noised bedgraph from running on original file; MACS2 peak calling run on AtacWorks output (9,350 total peaks in MACS2 file). b) Precision and Recall comparison of CTCF overlap between Chrom-Sig SICER peaks and AtacWorks MACS2 peaks, with Chrom-Sig result in green and AtacWorks result in yellow. c) Top enriched motifs, E-value, and matching motifs from MEME-ChIP for GM12878 CTCF ChIP-seq ENCFF500UFQ Chrom-Sig and AtacWorks.
